# What’s in a Name: A Bayesian Hierarchical Analysis of the Name-Letter Effect

**DOI:** 10.3389/fpsyg.2012.00334

**Published:** 2012-09-25

**Authors:** Oliver Dyjas, Raoul P. P. P. Grasman, Ruud Wetzels, Han L. J. van der Maas, Eric-Jan Wagenmakers

**Affiliations:** ^1^Department of Psychology, University of TübingenTübingen, Germany; ^2^Department of Psychology, University of AmsterdamAmsterdam, Netherlands

**Keywords:** analysis of large databases, Bayesian hierarchical hypothesis test, order-restrictions, random effects, name-letter effect

## Abstract

People generally prefer their initials to the other letters of the alphabet, a phenomenon known as the name-letter effect. This effect, researchers have argued, makes people move to certain cities, buy particular brands of consumer products, and choose particular professions (e.g., Angela moves to Los Angeles, Phil buys a Philips TV, and Dennis becomes a dentist). In order to establish such associations between people’s initials and their behavior, researchers typically carry out statistical analyses of large databases. Current methods of analysis ignore the hierarchical structure of the data, do not naturally handle order-restrictions, and are fundamentally incapable of confirming the null hypothesis. Here we outline a Bayesian hierarchical analysis that avoids these limitations and allows coherent inference both on the level of the individual and on the level of the group. To illustrate our method, we re-analyze two data sets that address the question of whether people are disproportionately likely to live in cities that resemble their name.

Social psychologists have claimed that the letters in a person’s name implicitly influence major life decisions such as where to live and what career to pursue. Concretely, this means that when your name is Louis the prospect of living in St. Louis is more attractive to you than to someone named Jim; that when your name is Denise you are more interested in dentistry than someone named Stacy; and that when your name is Tom you are more inclined to work for Toyota than when your name is Richard.

This arguably counterintuitive claim is supported by the statistical analysis of large databases (e.g., birth, marriage, and death records; telephone directories; memberships of professional organizations, and so forth; see Pelham et al., 2005, for a review, and see McCullough and McWilliams, 2010, 2011; LeBel and Paunonen, 2011; Pelham and Carvallo, 2011; Simonsohn, 2011a,b,c, for a critique and a discussion). For instance, in several studies using public records, Pelham and colleagues presented data suggesting that people are more likely to live in cities or states that resemble their first or last names (Pelham et al., 2002, Studies 1–5; Pelham et al., 2003, Study 1). Moreover, Pelham et al. (2002, Study 4) claimed that people move to states that resemble their names. In other studies, Pelham et al. (2002) showed that people’s names or initials predict whether they are dentists or lawyers (Study 7), or whether they are working in the hardware or the roofing business (Study 9).

In another attempt to demonstrate that people’s names influence major life decisions, Anseel and Duyck (2008) investigated whether people’s names are associated with the companies they work for (but see Simonsohn, 2011b). Anseel and Duyck (2008) sampled one third of all Belgian employes working in the private sector and indeed found that employes tend to work for companies whose initial letter matches their own. This effect was significant both across letters and for almost all letters individually (for other work on the effect of names on behavior see, e.g., Jones et al., 2004; Brendl et al., 2005; Nelson and Simmons, 2007; Chandler et al., 2008; see McCullough and McWilliams, 2010, 2011 for critiques on the Nelson and Simmons, 2007 study).

The most popular explanation for the above findings is “implicit egotism” (Pelham et al., 2002). According to this explanation, people have positive feelings about themselves. These positive feelings are associated automatically (i.e., implicitly, outside of conscious awareness) to places, events, and objects related to the self. Consistent with the above explanation, Nuttin (1985) first found that people tend to prefer the letters in their names to the other letters of the alphabet, a phenomenon known as the name-letter effect (henceforth NLE; Nuttin, 1987; Hoorens and Todorova, 1988; Hoorens et al., 1990; Greenwald and Banaji, 1995; Kitayama and Karasawa, 1997; Jones et al., 2002; but see Hodson and Olson, 2005).

Our goal here is not to debate whether it is plausible *a priori* that the NLE influences major life decisions; nor do we wish to evaluate the extent to which the NLE is caused by implicit egotism. Instead, our goal is to outline a new, Bayesian analysis to measure and judge the level of association between the letters of one’s name and major life decisions. Our Bayesian analysis is hierarchical, able to incorporate order-restrictions (i.e., the strong *a priori* expectation that the NLE is positive), and able to quantify evidence in support of the null hypothesis (e.g., Edwards et al., 1963; Gallistel, 2009; Rouder et al., 2009; Wetzels et al., 2009).

It is important to point out that recent work has identified several confounds that seriously compromise the conclusion from previous NLE analyses of large databases (e.g., McCullough and McWilliams, 2010, 2011; LeBel and Paunonen, 2011; Simonsohn, 2011a,b,c). Hence it may seem that our present methodological improvements amount to nothing more than rearranging the deck chairs on the Titanic.^1^ However, our purpose is much more general; we provide a tutorial-style exposition on the advantages of hierarchical Bayesian modeling, assessment of evidence using Bayes factors, and effective visualization of posterior distributions. The NLE discussion provides a case study that is useful to illustrate our main points – as will become clear later, previous debates in the NLE literature have centered around exactly those statistical problems that we can address through multi-level modeling. So despite the possible confounds, the NLE data are still useful because they illustrate the advantages of a general-purpose hierarchical Bayesian analysis.

The outline of this article is as follows. First, we describe two representative data sets (i.e., Pelham et al., 2002, Study 5 and Pelham et al., 2003, Study 1) and review the associated debate concerning the proper method of analysis. Second, we briefly introduce the fundamentals of Bayesian parameter estimation and hypothesis testing. Third, we present comprehensive Bayesian analyses for the two data sets and show by example the advantages of the Bayesian procedure over the procedures that are currently standard in the field.

## Data and Debate

As highlighted by the debate between Pelham et al. (2002, 2003) and Gallucci (2003), there is currently no generally accepted method for analyzing the impact of the NLE in large databases (see also Albers et al., 2009; LeBel and Gawronski, 2009; LeBel and Paunonen, 2011). For concreteness, we focus here on two examples and the subsequent debate about the correct method of data analysis. The first example is the *Saint city* data set (Pelham et al., 2002), which, according to Gallucci (2003), constitutes the most reliable data set from Pelham et al.’s (2002) original article. The second example is the *surname city* data set (Pelham et al., 2003). Both examples highlight the limitations and controversies that plague the standard methodologies, limitations and controversies that are subsequently addressed by our Bayesian hierarchical procedure.

### Example 1: The saint cities

In one of their archival studies, Pelham et al. (2002, Study 5) tested the notion that people gravitate toward cities that resemble their name. Specifically, Pelham et al. (2002) hypothesized that cities whose name begins with *Saint* followed by a person name (e.g., St. Louis, St. Paul) attract people who share that name (e.g., Louis, Paul) more than would be expected based on chance alone. To test this hypothesis, Pelham et al. (2002) considered all “Saint cities” in the U.S.; for each Saint city, they tabulated the proportion of deceased people with the matching Saint name (e.g., the proportion of people deceased in St. Louis named Louis). The authors then compared this proportion to the proportion of deceased people with the same name in the entire U.S. (e.g., the proportion of deceased people in the U.S. named Louis). With these data, it is possible to determine for example whether deceased residents of St. Louis were disproportionately likely to be named Louis, relative to all other Americans.

The original data appear in Table 1 (cf. Pelham et al., 2002, Table 8).^2^ The first column lists the names, the second column lists the proportion of deceased people in the entire U.S with that particular name, the third column lists the proportion of deceased people in the respective Saint city with the matching Saint name, and the fourth column lists the total number of people deceased in the respective Saint city, regardless of their name.

**Table 1:** **The Saint cities data set from Pelham et al., 2002, Table 8; male Saint names only)**.

	Name	Proportion of U.S. names	Proportion in city	City population	χ^2^	*p*
1.	Anthony	0.002508	0.003858	1,296	0.944	0.331
2.	Augustine*	0.000084	0.000000	13,057	1.097	0.295
3.	Bernard	0.001523	0.001600	1,250	0.005	0.944
4.	Charles	0.014408	0.015509	21,343	1.822	0.177
5.	David(s)*	0.004549	0.002035	2,948	4.115	0.043
6.	Elmo*	0.000126	0.000000	1,083	0.136	0.712
7.	**Francis**	0.002432	0.004752	2,315	5.136	0.023
8.	Gabriel*	0.000148	0.000000	276	0.041	0.840
9.	George*	0.014347	0.012532	6,942	1.617	0.203
10.	**Henry**	0.006720	0.033755	474	51.903	<0.001
11.	Ignace*	0.000007	0.000000	1,328	0.009	0.923
12.	**Jacob**	0.001111	0.005319	376	5.999	0.014
13.	James*	0.020204	0.015049	10,499	14.094	<0.001
14.	**Joe**	0.002471	0.005117	2,345	6.661	0.010
15.	John(s)*	0.029861	0.022749	5,187	9.057	0.003
16.	Joseph*	0.013665	0.008143	36,349	82.234	<0.0001
17.	Leonard	0.002038	0.002132	469	0.002	0.964
18.	**Louis**	0.004168	0.006206	358,699	358.942	<0.0001
19.	Mark(s)*	0.000679	0.000000	113	0.077	0.782
20.	Martin*	0.001477	0.000000	77	0.114	0.736
21.	Matthew(s)	0.000536	0.001037	1,928	0.903	0.342
22.	**Michael**	0.003717	0.013210	757	18.422	<0.0001
23.	Paul*	0.005469	0.005445	119,736	0.013	0.910
24.	Peter	0.002414	0.002956	2,706	0.330	0.566
25.	Stephen(s)*	0.001221	0.000549	1,823	0.675	0.411
26.	**Thomas**	0.007796	0.013746	873	3.996	0.046
27.	Vincent*	0.001080	0.000000	56	0.061	0.806

For each name, the table shows the proportion of deceased people in the U.S. with the Saint name (second column), the proportion of deceased people in the respective Saint city with the matching Saint name (third column), and the total number of people deceased in the respective Saint city (regardless of their name, fourth column). Names that failed to yield an effect in the predicted direction are marked with an asterisk (*). In addition to the data, χ^2^ values based on one degree of freedom and the associated *p*-values are also provided. Names that yielded a significant result (*p *< 0.05) are in boldface.

Calculation of the χ^2^ values. Let *e*_*i*_ denote the proportion of deceased people in the U.S. with a particular Saint name (i.e., second column). Analogously, let *o*_*i*_ denote the proportion of deceased people in the respective Saint city with the matching Saint name (i.e., third column). *TN*_*i*_ denotes the total number of people deceased in the respective Saint city, regardless of their name (i.e., fourth column). The χ^2^ values with *df *= 1 that appear in the fifth column are then calculated as follows: $\chi_{i}^{2}=TN_{i}{\left(o_{i}-e_{i}\right)}^{2}∕e_{i}+TN_{i}{\left(\left(1-{\text{o}}_{\text{i}})-(1-e_{i}\right)\right)}^{2}∕\left(1-e_{i}\right)$

*[cf. Lewis and Burke, 1949, Equation (6a)]*.

### Previous analysis and criticism

In their article “Why Susie sells seashells by the seashore: Implicit egotism and major life decisions,” Pelham et al. (2002, p. 476) report “On the basis of expected values, 3,476.0 [*sic*] out of 594,305 men should have lived in Saint cities bearing their first names. The actual number of men who did so was 3,956, which is 14% greater than the chance value. Because of the extremely large sample size for men, this value was also highly significant, χ^2^(1) = 58.63, *p* < .001.” From these results, the authors conclude that the NLE influences where people choose to live. The statistical test with one degree of freedom is based on two comparisons, namely the expected versus observed frequency of people who deceased in cities that resembled their names (matches) and the expected versus observed frequency of people who did *not* decease in cities that resembled their names (mismatches), both pooled across all cities (see Pelham et al., 2003, p. 800, for a comment on their original analysis).

In his article “I sell seashells by the seashore and my name is Jack: Comment on Pelham, Mirenberg, and Jones (2002),” Gallucci (2003) criticized the way Pelham et al. (2002) had analyzed their data. Specifically, Gallucci pointed out that the overall test ignores the fact that the units of analysis are individual names and cities; the data are nested, with individuals nested under names and cities. Gallucci remarked that the overall test from Pelham and colleagues might yield a significant result due to a single outlying city.

Instead of the complete pooling analysis used by Pelham and colleagues, Gallucci (2003, p. 790) advocated a complete independence approach: “The correct test of the hypothesis should generalize the effect across names. We therefore need to test how many names reveal a significant effect in support of the hypothesis, how many are not in support of the hypothesis, and how many, if any, are against the hypothesis (i.e., significantly less than chance).” Thus, Gallucci (2003) sought to test Pelham et al.’s (2002) hypothesis on a name-by-name basis. To do so, Gallucci conducted χ^2^ tests with one degree of freedom for each name separately and counted the number of significant results. Table 1, last two columns, lists the χ^2^ test statistics with one degree of freedom and the corresponding *p*-values for each name. Note that some of our numbers differ slightly from those reported by Gallucci (2003, Table 1). Gallucci (2003) found that out of the 27 name-city matches under consideration, 10 were significantly different from chance (11 in our calculations). However, only 6 (7 in our calculations) were in the expected direction. In other words, in 4 Saint cities, *fewer* people with that name deceased than one would expect by chance (i.e., a reverse NLE). Gallucci (2003) considers these 4 Saint cities as evidence against Pelham et al.’s (2002) key hypothesis. Moreover, Gallucci (2003) argues that if just one Saint city – Saint Louis – is left out of the overall analysis, the overall result is in the opposite direction from the NLE hypothesis (i.e., without Saint Louis, the observed frequency of name matches is 1,729 and the expected frequency is 1,981). Gallucci therefore concluded that the original results in support of Pelham et al.’s (2002) hypothesis originate from just one supportive Saint city, namely Saint Louis.

In their rejoinder paper, Pelham et al. (2003) argued that the name-by-name analysis suggested by Gallucci (2003) is only appropriate for large cities, when name-city combinations yield large expected frequencies. According to Pelham and colleagues, it would be unfair to assign equal weight to a small city such as Saint Gabriel and a large city such as Saint Louis.

In sum, Gallucci (2003) advocated complete independence, whereas Pelham et al. (2002) advocated complete pooling. As we illustrate later, in between these two extremes lies the compromise of Bayesian hierarchical modeling, in which the individual differences between cities are restricted by group-level information (Gelman and Hill, 2007; Lee, 2011).

### Example 2: The surname cities

Along with their rejoinder commentary, Pelham et al. (2003) presented additional archival studies in support of their implicit egotism hypothesis. In one of these studies, Pelham et al. (2003, Study 1) tested the notion that people gravitate toward cities whose names include these people’s complete surnames (e.g., Johnsonville or Johnson City). To test this hypothesis, Pelham and colleagues considered the 30 most common European American surnames in the U.S.; as in the Saint cities study, Pelham and colleagues then collected the proportion of people with that surname deceased in the respective surname city (e.g., the proportion of people named Johnson deceased in Johnsonville) and the proportion of people with that surname deceased in the entire U.S. (e.g., the proportion of people named Johnson deceased in the entire U.S.). The data appear in Table 2 (cf. Pelham et al., 2003, Table 1).

**Table 2:** **The surname cities data set from Pelham et al., 2003, Table 1)**.

	Surname	Proportion of U.S. surnames	Proportion in city resembling name	City population	χ^2^	*p*
1.	**Smith**	0.01000	0.01235	66,582	37.141	<0.0001
2.	Johnson*	0.00749	0.00688	31,532	1.578	0.209
3.	**Williams**	0.00574	0.00636	74,218	4.999	0.025
4.	**Jones**	0.00546	0.00897	36,576	82.984	<0.0001
5.	Brown	0.00558	0.00635	28,201	3.013	0.083
6.	**Davis**	0.00433	0.00899	14,133	71.187	<0.0001
7.	**Miller**	0.00485	0.01949	13,956	619.745	<0.0001
8.	Wilson	0.00328	0.00372	22,017	1.304	0.254
9.	Moore	0.00292	0.00352	13,358	1.652	0.199
10.	Taylor	0.00291	0.00311	31,228	0.431	0.512
11.	Anderson	0.00336	0.00374	51,346	2.214	0.137
12.	**Thomas**	0.00267	0.00537	44,540	121.935	<0.0001
13.	**Jackson**	0.00248	0.00433	320,516	443.425	<0.0001
14.	**White**	0.00265	0.00321	103,055	12.228	<0.001
15.	Harris	0.00236	0.00244	88,932	0.242	0.623
16.	**Martin**	0.00273	0.00408	37,511	25.110	<0.0001
17.	Thompson	0.00266	0.00292	5,132	0.131	0.718
18.	Robinson	0.00192	0.00260	5,002	1.207	0.272
19.	Clark	0.00232	0.00257	52,625	1.421	0.233
20.	Lewis	0.00204	0.00205	66,431	0.003	0.954
21.	Lee	0.00165	0.00181	43,574	0.677	0.411
22.	Walker	0.00201	0.00243	8,231	0.724	0.395
23.	Hall	0.00196	0.00254	20,112	3.459	0.063
24.	Allen*	0.00191	0.00158	71,320	4.074	0.044
25.	Young	0.00191	0.00204	72,163	0.640	0.424
26.	King	0.00181	0.00190	120,402	0.540	0.463
27.	**Wright**	0.00176	0.00459	3,485	15.886	<0.0001
28.	Hill*	0.00174	0.00157	386,905	6.437	0.011
29.	Scott	0.00169	0.00184	58,039	0.774	0.379
30.	Green	0.00169	0.00176	386,920	1.124	0.289

For each surname, the table shows the proportion of people with that name deceased in the U.S. (second column), the proportion of people with that name deceased in the respective surname city (third column), and the total number of people deceased in the respective surname city (regardless of their name, fourth column). As in the original table, surnames with a reverse NLE (no matter how small) are marked with an asterisk (*). Columns five and six provide χ^2^ values based on one degree of freedom and their associated *p*-values. Names that yielded a significant result (*p *< 0.05) are in boldface.

In their analysis of the surname cities data, Pelham et al. (2003) treated surnames as the units of analysis in a matched-samples *t*-test. For each of the 30 surnames, there were two observations: the proportion of people with that surname deceased in the respective surname city (e.g., the proportion of people named Johnson deceased in Johnsonville) and the proportion of people with that surname deceased in the U.S. Pelham et al. (2003, p. 803) reported a significant result, *t*(29) = 2.58, *p* = 0.015 and concluded that “(…) implicit egotism is a highly robust phenomenon.” For the sake of comparability, we calculated χ^2^ tests with one degree of freedom for each surname separately, just as Gallucci (2003) did for the Saint cities. The last two columns of Table 2 list the χ^2^ test statistics with one degree of freedom and the corresponding *p*-values for each surname. From the 30 possible name-city matches, 12 were significantly different from chance, *p* < 0.05. From these 12 significant matches, all but two – namely Allen (no. 24) and Hill (no. 28) – were in support of the NLE hypothesis; in other words, for 10 surnames significantly more people with that surname deceased in the surname-resembling city than one would expect based on statistics of the U.S. population.

### Interim conclusion

Both prevalent methods for analyzing NLE data can be criticized. Pelham et al. (2002) used complete pooling and calculated an overall test, ignoring the fact that the cities may differ from each other. Gallucci (2003) assumed complete independence and calculated a χ^2^ statistic and an associated *p*-value for each name separately. This test ignores the fact that the cities may be similar to each other.

In the remainder of this article we propose an alternative, Bayesian method for the analysis of the NLE in large databases. Our Bayesian method accounts for the hierarchical structure of the data and hence incorporates both the differences and the similarities between cities. Before we outline our Bayesian method, however, we briefly introduce Bayesian parameter estimation and hypothesis testing. The reader who is familiar with these concepts can safely skip to the next section.

## Basics of Bayesian Inference

This section provides a short overview of Bayesian inference. More detailed information can be found in Bayesian articles and books that discuss philosophical foundations (Lindley, 2000; O’Hagan and Forster, 2004), computational innovations (Gamerman and Lopes, 2006), and practical contributions (Congdon, 2003; Ntzoufras, 2009). Recent introductions for psychologists are given for instance by Hoijtink et al. (2008), Kruschke (2010a,b), Lee and Wagenmakers (to appear), and Wagenmakers et al. (2010).

### Bayesian parameter estimation

In Bayesian inference, parameters are random variables. Uncertainty or degree of belief about the parameters is quantified by probability distributions. For a particular model that contains a parameter δ, the observed data *D* update a prior distribution *p*(δ)according to Bayes’ rule to yield a posterior distribution *p*(δ|*D*). The prior distribution for δ reflects our knowledge about δ before observing data *D*, and the posterior distribution for δ reflects our knowledge about δ after observing data *D*. Specifically, Bayes rule shows that the posterior distribution *p*(δ|*D*) is equal to the product of the prior *p*(δ)and the likelihood *p*(*D*|δ), divided by the marginal likelihood *p*(*D*).

(1) $$\text{Posterior}=\frac{\text{prior}\times \text{likelihood}}{\text{marginal}\;\text{likelihood}}.$$

Or, expressed symbolically:

(2) $$p(\delta |D)=\frac{p(\delta )\times p(D|\delta )}{p(D)}.$$

The marginal likelihood *p*(D) is a single number, a normalizing constant that ensures that the posterior distribution has area 1. Hence, *p*(D) is not essential for parameter estimation, and one can simplify the above relation by stating that the posterior distribution is proportional to (i.e., ∝) the prior times the likelihood:

(3) p(δ|D)∝p(δ)×p(D|δ).

For parameter estimation, the specific shape of the prior distribution is often not very influential; with the relatively large amount of data available in most psychological experiments, prior distributions that are very different nevertheless yield posterior distributions that are almost identical. Intuitively, this happens because the posterior distribution is a rational compromise between the information we had before we encountered the data (i.e., the prior), and the information provided by the data themselves (i.e., the likelihood) – as formalized by equation (3). Hence it is said that the data overwhelm the prior (e.g., Lee and Wagenmakers, 2005). Thus, rational people with widely different prior beliefs will ultimately converge to the same posterior beliefs.

For many models, the posterior distribution cannot be obtained analytically. In such cases, one can use Markov chain Monte Carlo (MCMC) techniques to draw consecutive samples from the posterior distribution – by plotting these samples as a histogram, this numerical method allows one to approximate the posterior distribution to any desired degree of accuracy. In this article we conducted MCMC sampling with the widely used WinBUGS software program (i.e., Bayesian inference Using Gibbs Sampling^3^; Lunn et al., 2000, 2009). WinBUGS is designed so that the user can specify and fit complex statistical models without having to hand-code the MCMC algorithms. The Appendix shows how our model for the name-letter effect in large databases can be represented in a few lines of easy-to-understand WinBUGS code.

Note that whenever one uses an MCMC method it is important to ascertain that the sequence of samples (i.e., a *chain*) has lost its dependence on the starting value such that the samples are indeed draws from the posterior distribution. Using different chains, each with a different “overdispersed” starting value, one can confirm convergence to the posterior using visual inspection^4^ and statistics such as $\hat{\text{R}}$ (Gelman and Rubin, 1992).

One of the practical advantages of Bayesian inference is that it allows for the flexible implementation of relatively complicated statistical techniques such as those that involve hierarchical non-linear models. In hierarchical Bayesian models, one usually starts by assuming that individual-level parameters are constrained by a Gaussian group distribution, *N*(μ, σ); because σ corresponds to the spread of the group distribution, this parameter quantifies the extend to which the individual units differ – low values of σ indicate that the units are relatively similar; in the limit of σ → 0, all units are identical copies of each other. The theoretical advantages and practical relevance of a Bayesian hierarchical analysis for common experimental designs have been repeatedly demonstrated by Jeff Rouder and colleagues (e.g., Rouder and Lu, 2005; Rouder et al., 2005, 2007, 2008; see also Shiffrin et al., 2008; Lee, 2011; Nilsson et al., 2011; van Ravenzwaaij et al., 2011). One of the theoretical advantages is that by hierarchical modeling, researchers automatically obtain an optimal compromise between the extremes of complete pooling and complete independence. One of the practical advantages is that hierarchical modeling allows for more efficient inference on the individual level; this happens because extreme individual estimates, when these are based on few data, are shrunk toward the group mean (Gelman and Hill, 2007).

After fitting a Bayesian hierarchical model to data, posterior distributions quantify uncertainty both on the level of the individual unit and on the level of the group.

### Bayesian hypothesis testing

In psychological research, competing hypotheses are often formulated as nested models. The null hypothesis *H*_0_ states that a particular effect is absent, such that the corresponding parameter equals zero, that is, *H*_0_ : δ = 0. The alternative hypothesis *H*_1_ is usually not specified exactly and states that the effect is present, that is, *H*_1_ : δ ≠ 0.^5^ In psychological practice, hypothesis testing proceeds by calculating a *p*-value, rejecting *H*_0_ when *p* < 0.05 and “failing to reject” *H*_0_ otherwise.

In contrast to popular *p*-value practice, Bayesian hypothesis testing seeks to quantify the relative plausibility of *H*_0_ and *H*_1_ (Wagenmakers and Grünwald, 2006; Gallistel, 2009; Rouder et al., 2009; for recent discussions see Wagenmakers et al., 2011; Wetzels et al., 2011). As in parameter estimation, one starts by assigning prior probability to *H*_0_ and *H*_1_; the prior model odds [i.e., *p*(*H*_0_)/*p*(*H*_1_)] is then updated through the data *D* to yield the posterior model odds [i.e., *p*(*H*_0_|*D*)/*p*(*H*_1_|*D*)]. The change from prior to posterior model odds, brought about by the observed data, is called the *Bayes factor* (Jeffreys, 1961; Kass and Raftery, 1995):

(4) $$\text{Bayes}\;\text{factor}=\frac{\text{posterior}\;\text{odds}}{\text{prior}\;\text{odds}}=\frac{\text{odds}\left(H_{0}\;\text{vs}.\;H_{1}|\text{observed}\;\text{data}\right)}{\text{odds}\left(H_{\text{0}}\;\text{vs}.\;H_{1}\right)}$$

When the Bayes factor *BF*_01_ for model *H*_0_ versus *H*_1_ equals 2, this means that the data are twice as likely to have occurred under *H*_0_ than under *H*_1_. Thus, a hypothesis test based on the Bayes factor prefers the model under which the observed data are most likely. As such, the Bayes factor represents “the standard Bayesian solution to the hypothesis testing and model selection problems” (Lewis and Raftery, 1997, p. 648).

In this article we compute Bayes factors using the so-called *Savage–Dickey density ratio*. Consider our example above, where the null hypothesis *H*_0_ : δ = 0 is nested in the alternative hypothesis *H*_1_ : δ ≠ 0. When *H*_0_ is nested in *H*_1_, the Savage–Dickey density ratio states that the Bayes factor can be determined by considering only the posterior and prior distributions for parameter δ in *H*_1_, evaluated at the value that is subject to test (e.g., Verdinelli and Wasserman, 1995; O’Hagan and Forster, 2004, pp. 174–177; Gamerman and Lopes, 2006, pp. 72–74, pp. 79–80; Wetzels et al., 2009, 2010; Wagenmakers et al., 2010). To illustrate, when the posterior distribution for δ (under *H*_1_) has height 3 at δ = 0, and the prior distribution for δ (under *H*_1_) has height 1 at δ = 0, then the data are three times more likely to have occurred under *H*_0_ then under *H*_1_. Thus

(5) $$BF_{01}=\frac{p\left(D|H_{0}\right)}{p\left(D|H_{1}\right)}=\frac{p\left(\delta =0|D,H_{1}\right)}{p\left(\delta =0|H_{1}\right)}.$$

Compared to most alternative methods, the Savage–Dickey density ratio allows for a relatively simple and intuitive assessment of the Bayes factor. It should be stressed, however, that – in contrast to Bayesian parameter estimation – the Bayes factor remains sensitive to the prior distribution for the parameter δ that is subject to test, even after a considerable amount of data has been collected. Thus, in the case of Bayesian hypothesis testing, the data do not overwhelm the prior. It is therefore essential that particular attention is paid to the prior distribution for the parameter that is subject to test. In general, it is good practice to carry out both parameter estimation (for which the data quickly overwhelm the prior) and hypothesis testing (for which the prior has a lasting impact). In many situations the conclusions that are drawn will be qualitatively the same: when the posterior distribution for δ is far away from 0 the Bayes factor indicates strong support for *H*_1_. We now apply Bayesian parameter estimation and hypothesis testing to the data sets discussed previously.

## Bayesian Hierarchical Analysis in Practice

Recall the two studies testing the notion that people gravitate toward cities that resemble their name. In both studies, Pelham and colleagues sampled a number of names and a number of cities with similar names. For each name, they then compared the proportion of people with that name deceased in the respective city to the proportion of people with that name deceased in the U.S. In our first example, this comparison was done for cities whose names begin with Saint, followed by a person name. In our second example, the comparison was done for cities whose names include complete surnames. Tables 1 and 2 list the Saint city and the surname city data set, respectively.

### Bayesian analysis for example 1: The saint cities

Let *N*_*i*_ denote the number of people with name *i* who died in Saint city *i* (i.e., the number of people named Louis who died in St. Louis), and let *TN*_*i*_ denote the total number of people who died in Saint city *i*, regardless of their name. For each name *i*, we assumed that *N*_*i*_ out of *TN*_*i*_ is binomially distributed with rate parameter *θ*_*i*_. To assess whether there is a NLE and *θ*_*i*_ is disproportionally large, we need to compare *θ*_*i*_ to what can be expected in the entire U.S. At first sight, it may seem reasonable to quantify the NLE for city *i* by *θ*_*i*_ − *b*_*i*_, where *b*_*i*_ is the baseline proportion of people with name *i* deceased in the U.S.

Unfortunately, both *θ*_*i*_ and *b*_*i*_ are defined on the rate scale, which ranges from 0 to 1 and is not suitable for modeling additive effects. We therefore first transformed *θ*_*i*_ to γ_*i*_ and *b*_*i*_ to β_*i*_ using the probit transformation. The probit transform maps probabilities into *z*-values using the inverse cumulative distribution function of the standard Normal distribution. In contrast to the rate scale, the probit scale ranges across the entire real number line and is appropriate for modeling additive effects (Rouder and Lu, 2005).

Thus, we obtain the NLE for name *i*, *α*_*i*_, by subtracting the probitized U.S. baseline of occurrence from the probitized city-specific rate of occurrence, that is, α_*i*_ = γ_*i*_ − β_*i*_. Hence, positive values for α are in line with Pelham et al.’s (2002) hypothesis and indicate, for instance, that more people named Louis deceased in St. Louis than one would expect from the American population. In contrast, negative values for α indicate a reverse NLE, that is for instance, fewer people named Louis deceased in St. Louis than one would expect.

**Figure 1:**
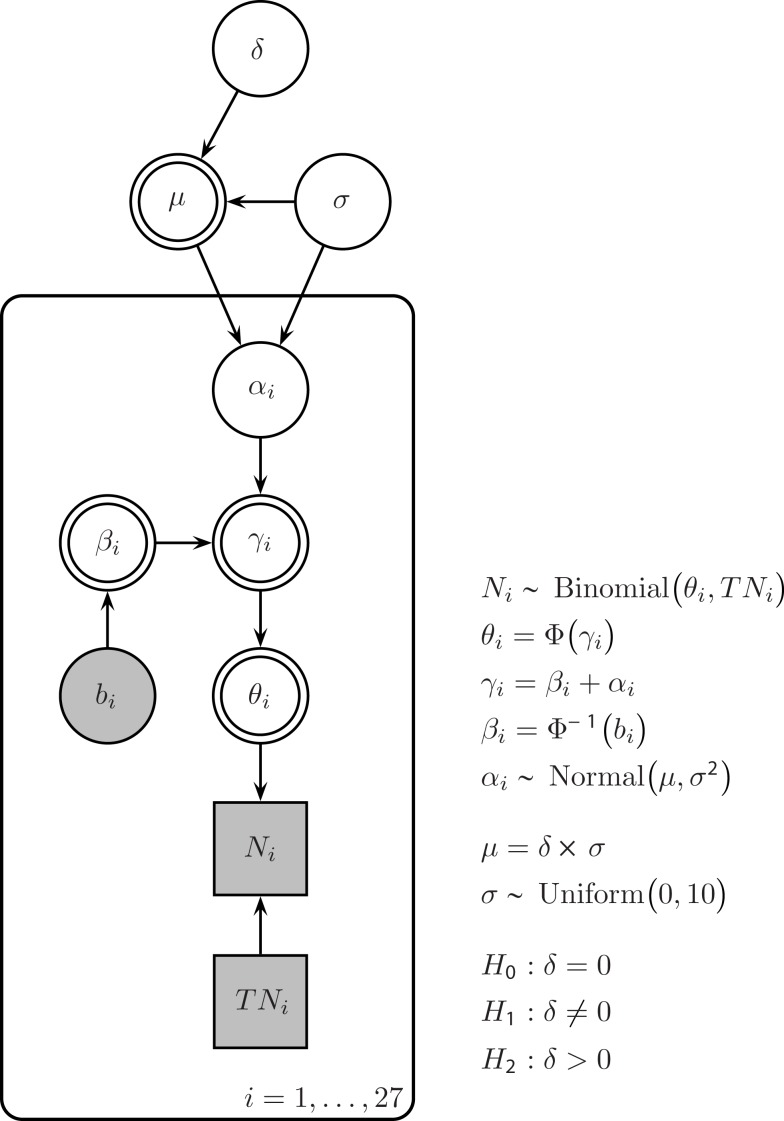
**Bayesian graphical model for the Saint cities data**. Parameter α_*i*_ quantifies the name-letter effect for city *i*, that is, the difference between the probit-transformed U.S. population rate β_*i*_ and the inferred probit-transformed rate for city *i*. In turn, α_*i*_ is modeled as a random effect, that is, it is governed by a group-level Normal distribution with mean μ and SD σ. The plate indicates that this group-level structure holds for all *i* = 1,…, 27 cities. Effect size δ is defined as μ/σ, and the prior on δ is a standard Normal.

Figure 1 shows our model for the Saint cities data in standard graphical model notation (e.g., Gilks et al., 1994; Lunn et al., 2000; Lee and Wagenmakers, to appear). In this notation, nodes represent variables and the dependency of these variables is indicated by arrows with children depending on their parents. Circular nodes represent continuous variables (e.g., rate θ), and square nodes represent discrete variables (e.g., number of people *TN*_*i*_). Observed variables are shaded (and denoted by Latin letters, e.g., baseline proportion *b*_*i*_ for city *i*) and unobserved variables are not shaded (and denoted by Greek letters, e.g., inferred name-letter effect α_*i*_ for city *i*). Double borders indicate that the variable is deterministic (i.e., calculated without noise from other variables, e.g., γ_*i*_ is given by β_*i*_ + α_*i*_) rather than stochastic.

It has been argued that the NLE is not the same for every name. For instance, the NLE may differ due to the frequency of the name (e.g., disappear “for extremely common and thus less self-defining male first names,” Pelham et al. (2003, p. 802) or due to the size of the Saint city: “Implicit egotism should be stronger for rare rather than common names. Rare names tend to generate small sample sizes.” Pelham et al. (2003, p. 802). Consequently, rather than assuming that the NLE is a fixed effect, we assume that it is a random effect. Specifically, we assumed that an individual α_*i*_ is drawn from a group-level Gaussian distribution with mean μ and SD σ. The hierarchical aspect of our model is indicated in Figure 1 by the plate that encloses subsets of the graph that have independent replications.

Because our analysis is Bayesian, the group-level parameters μ and σ require prior distributions. For the SD σ of the group-level distribution, we chose an uninformative uniform prior from 0 to 10. Instead of assigning a prior to μ we assigned a prior to the effect size δ = μ/σ. Effect size is a dimensionless quantity that applies across different studies. Therefore, for effect size, a principled default prior is relatively easy to define. One uninformative or objective prior on effect size is the standard Normal distribution (Rouder et al., 2009). This prior is known as the “unit information prior” and carries as much information as a single observation (Kass and Wasserman, 1995).

The computational implementation of our model and the details of the MCMC sampling are described in the Appendix. The results below are based on 150,000 draws from the joint posterior distribution.

#### Parameter estimation: NLEs for individual names

**Figure 2:**
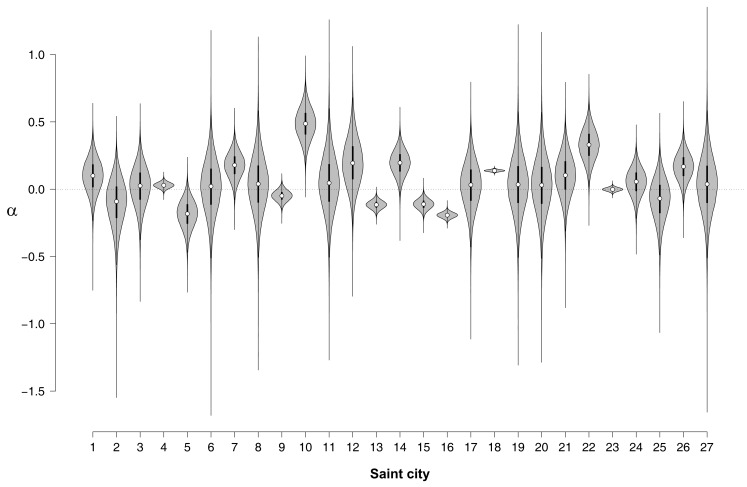
**Violin plot of the posterior distributions of α for each of the Saint cities**. Note that the distributions do not integrate to 1 in this representation. 1, Anthony; 2, Augustine; 3, Bernard; 4, Charles; 5, David(s); 6, Elmo; 7, Francis; 8, Gabriel; 9, George; 10, Henry; 11, Ignace; 12, Jacob; 13, James; 14, Joe; 15, John(s); 16, Joseph; 17, Leonard; 18, Louis; 19, Mark(s); 20, Martin; 21, Matthew(s); 22, Michael; 23, Paul; 24, Peter; 25, Stephen(s); 26, Thomas; 27, Vincent.

In order to assess the NLE for individual names, Figure 2 shows violin plots (Hintze and Nelson, 1998) for the posterior distributions of α. A violin plot combines box plot and density trace. The box plot part shows center, spread, and asymmetry of a variable, where a circle marks the median and the bounds of the box indicate the first and third quartile. The density trace is plotted symmetrically to the left and right of the vertical box plot, making it easier to see the magnitude of the density.

Figure 2 shows that for Saint Henry (city no. 10) the median of the NLE is clearly above zero, indicating that more people named Henry deceased in Saint Henry than one would expect from the U.S. base rate. A positive NLE is also observed for Saint Louis (city no. 18), a relatively large city for which the NLE can be estimated precisely; this high precision is reflected in the small spread of the posterior distribution for α_18_.

Figure 2 also shows that for Saint Elmo (city no. 6), the median of the NLE is approximately zero, although there is substantial uncertainty about this estimate; for Saint Paul (city no. 23), the median of the NLE is also approximately zero, but this estimate has relatively little uncertainty. Finally, Figure 2 also suggests that a few names show a reliable *reverse* NLE; for instance, in the case of Saint Joseph (city no. 16), the median is clearly below zero. The finding of a reverse NLE is not easily accommodated by current theories of how implicit egotism influences major life decisions.

Although informative, Figure 2 does not allow a precise assessment of the presence of a positive NLE on the group-level. Some names show a positive NLE, some names show a negative effect, and many names do not allow a definitive judgment. To quantify the evidence for and against the NLE on the group-level we now turn to a Bayes factor hypothesis test.

#### Hypothesis testing: unrestricted analysis

Even though Figure 2 allows a detailed assessment of the NLE on the level of each individual name or city, we have not yet combined this information to make a group-level judgment on the plausibility of the NLE. In order to do so, we contrast two hypotheses with respect to the group-level effect size δ. The first hypothesis is the null hypothesis and it states that there is no overall NLE; hence, the effect size is zero, *H*_0_ : δ = 0. The alternative, unrestricted hypothesis states that there is an overall NLE, which might be positive, as hypothesized by Pelham et al. (2002), or “reverse”; hence, the effect size is free to vary, *H*_1_ : δ ≠ 0. As mentioned above, the Bayesian analysis necessitates that one is precise about the prior for δ under *H*_1_, and here we make use of the default standard Normal prior: *p*(δ) ∼ *N*(0,1).

**Figure 3:**
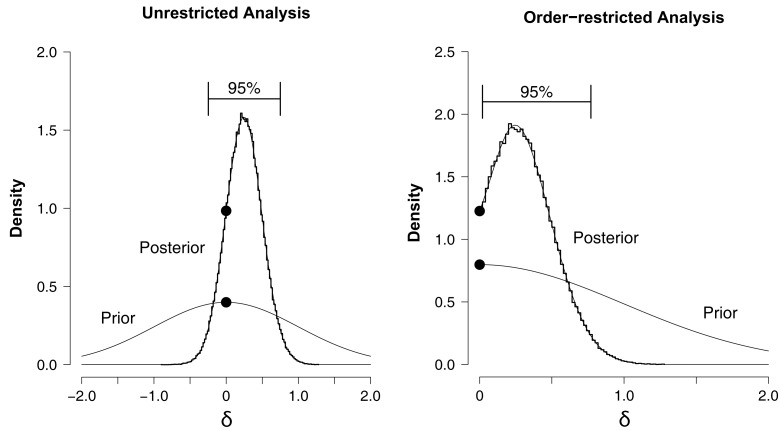
**Prior and posterior distributions of the group-level effect size δ for the hierarchical analysis of the Saint cities data set**. Left panel: unrestricted analysis, right panel: order-restricted analysis. The prior distribution is the standard Normal (thin line). The posterior distribution is indicated by a histogram of MCMC samples (thick line) and the corresponding logspline non-parametric density estimate (thin line). The 95% confidence interval for the posterior extends from −0.247 to 0.750 for the unrestricted analysis (left panel), and from 0.020 to 0.770 for the order-restricted analysis (right panel). The black dots mark the height of the prior and the posterior at the point of interest δ = 0.

The left panel of Figure 3 shows the prior and posterior distributions for effect size parameter δ under *H*_1_.^6^ Although most of the distribution lies to the right of zero, the 95% confidence interval ranges from −0.247 to 0.750 and overlaps with zero.^7^ The two dots mark the height of the prior and posterior distribution at the point of interest δ = 0, obtained from a logspline non-parametric density estimate (Stone et al., 1997). According to the Savage–Dickey density ratio *BF*_01_ ≈ 2.47, which means that the data are about 2.47 times more likely under the null hypothesis *H*_0_ than under the unrestricted alternative *H*_1_. In sum, the unrestricted analysis suggests that there is no overall NLE, although the evidence is not strong.

#### Hypothesis testing: order-restricted analysis

In the unrestricted analysis, we tested whether δ ≠ 0. However, Pelham et al.’s (2002) hypothesis was more specific: cities should attract and not deter people with the same name. Thus, the hypothesis of an overall NLE can be recast as δ > 0. Hence, our order-restricted analysis tests: *H*_0_ : δ = 0 versus *H*_2_ : δ > 0.

We implemented this order-restriction in two ways. The first method is based on renormalization, dividing the height of the unrestricted posterior at δ = 0 by the area to the right of δ = 0. The same is done for the height of the prior. The ratio of these renormalized heights is then the Bayes factor for *H*_0_ versus the order-restricted *H*_2_. This method resulted in *BF*_02.*M*1_ ≈ 1.48. In the second method one discards the MCMC samples that are inconsistent with the order-restriction. The remaining samples that obey the order-restriction are then used to plot histograms and construct a density estimate. The procedure is otherwise the same as described for the unrestricted analysis. This method resulted in *BF*_02.*M*2_ ≈ 1.54. This is visualized in the right panel of Figure 3. Thus, both methods indicate that the data are about 1.5 times more likely under the null hypothesis *H*_0_ than under the order-restricted alternative *H*_2_. This evidence in favor of the null is slightly weaker than it was in the unrestricted analysis.

In sum, our hierarchical Bayesian analysis of the Saint city data provided no support for the hypothesis that people gravitate to cities that resemble their name. In fact, our analysis provided some arguably weak support in favor of the null hypothesis.

### Bayesian analysis for example 2: The surname cities

The structure of the surname cities data set is equivalent to the Saint cities data set. Therefore, we used the same model and the same analysis procedure. As before, the results are based on 150,000 draws from the joint posterior distribution (see Appendix for details).

#### Parameter estimation: NLEs for individual names

**Figure 4:**
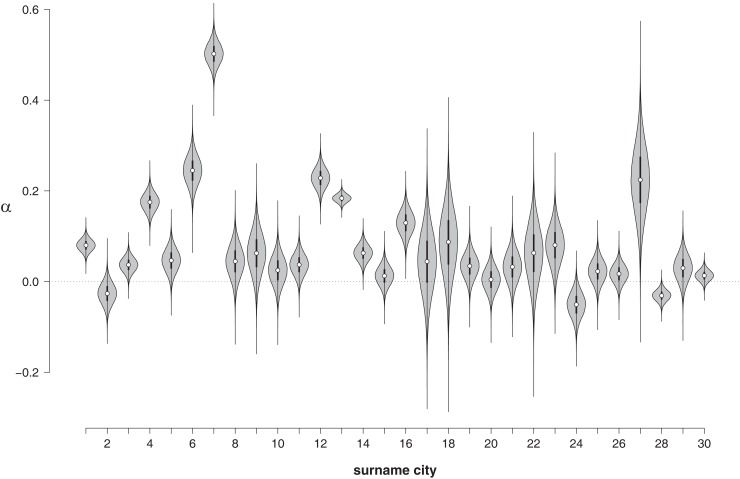
**Violin plot of the posterior distributions of α for each of the surname cities**. Note that the distributions do not integrate to 1 in this representation. 1, Smith; 2, Johnson; 3, Williams; 4, Jones; 5, Brown; 6, Davis; 7, Miller; 8, Wilson; 9, Moore; 10, Taylor; 11, Anderson; 12, Thomas; 13, Jackson; 14, White; 15, Harris; 16, Martin; 17, Thompson; 18, Robinson; 19, Clark; 20, Lewis; 21, Lee; 22, Walker; 23, Hall; 24, Allen; 25, Young; 26, King; 27, Wright; 28, Hill; 29, Scott; 30, Green.

In order to assess the NLE for individual names, Figure 4 shows the posterior distribution α_*i*_ for each surname city as a violin plot. For most names, the median of the posterior distribution is clearly above zero. The NLE seems to be particularly pronounced for Miller (city no. 7). For only three out of 30 cities (Johnson, no. 2; Allen, no. 24; Hill, no. 28) is there a clear indication of a reverse NLE. Hence, Figure 4 suggests that the surname city data set may indeed show a positive NLE on the group-level. To quantify the evidence for and against the NLE on the group-level more precisely we again turn to a Bayes factor hypothesis test.

#### Hypothesis testing: unrestricted analysis

As for the Saint cities data set, we again contrast two hypotheses with respect to the group-level effect size δ. The first hypothesis is the null hypothesis and it states that there is no overall NLE; hence, the effect size is zero, *H*_0_ : δ = 0. The unrestricted alternative hypothesis states that the effect size is free to vary, *H*_1_ : δ ≠ 0; again, we assigned δ a standard Normal prior distribution.

**Figure 5:**
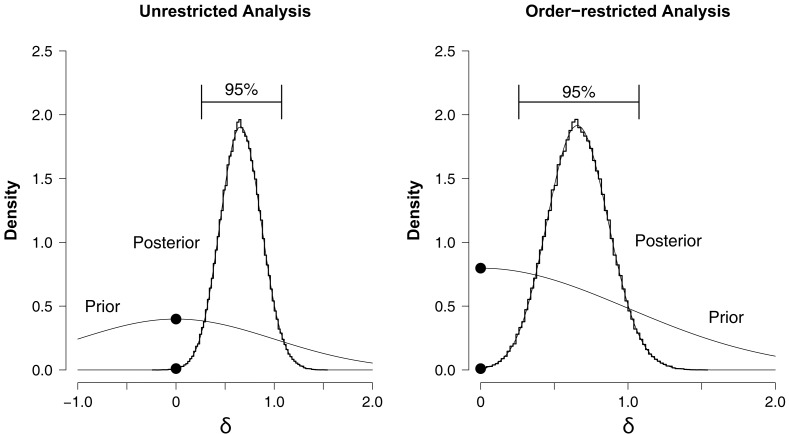
**Prior and posterior distributions of the group-level effect size δ for the hierarchical analysis of the surname cities data set**. Left panel: unrestricted analysis, right panel: order-restricted analysis. For the prior, the distribution is shown (thin line). For the posterior, a histogram (thick line) and the logspline non-parametric density estimate (thin line) are depicted. The 95% confidence interval for the posterior extends from 0.260 to 1.075 for the unrestricted analysis (left panel), and from 0.259 to 1.075 for the order-restricted analysis (right panel). The black dots tag the height of the prior and the posterior at the point of interest δ = 0.

The left panel of Figure 5 shows the prior and posterior distribution for effect size δ under *H*_1_. For the posterior distribution, almost all of the mass lies to the right of zero and the 95% confidence interval (i.e., 0.260–1.075) does not overlap with zero. The two dots mark the height of the prior and posterior distribution at the point of interest δ = 0, and, according to the Savage–Dickey density ratio, *BF*_01_ ≈ 0.03, which means that the data are about *BF*_10_ = 1/*BF*_01_ ≈ 37.23 times more likely under the unrestricted alternative *H*_1_ than they are under the null hypothesis *H*_0_. In sum, the unrestricted analysis suggests that there is indeed an overall NLE for the surname cities data.

#### Hypothesis testing: order-restricted analysis

The order-restricted analysis tests the notion that people gravitate toward and not away from cities whose names include their own (Pelham et al., 2003). That is, the order-restricted analysis tests *H*_0_ : δ = 0 versus *H*_2_ : δ > 0.

We again used two methods to compute the Bayes factor for the order-restricted test. The first method uses renormalization and yielded *BF*_02.*M*1_ ≈ 0.01 in favor of the null hypothesis, or – equivalently – *BF*_20.*M*1_ ≈ 74.42 in favor of the order-restricted alternative hypothesis. The second method uses only the samples that obey the order-restriction and yielded *BF*_02.*M*2_ ≈ 0.01 in favor of the null hypothesis or *BF*_20.*M*2_ ≈ 77.45 in favor of the order-restricted alternative hypothesis. This result is visualized in the right panel of Figure 5. Thus, both methods indicate that the data are about 75 times more likely under the order-restricted alternative *H*_2_ than under the null hypothesis *H*_0_. The evidence in favor of the alternative hypothesis is about twice as strong as it was in the unrestricted analysis.

In sum, our Bayesian hierarchical analysis of the surname cities data set clearly indicated the presence of an overall NLE: the data are about 75 times more likely to have occurred under *H*_2_, the hypothesis that the group-level effect size δ is greater than zero, than under *H*_0_, the hypothesis that δ is equal to zero. Simonsohn (2011c) recently suggested that this effect is spurious, because “a staggering number of towns containing a last name in their name were founded by individuals with such last names.” The evaluation of whether or not the NLE is entirely produced by confounds is beyond the scope of this paper – here we merely quantify the statistical evidence for and against the presence of the effect, without recourse to its possible cause. This is consistent with the main purpose of this paper, which is to provide a tutorial-style introduction to the advantages of hierarchical Bayesian modeling, the assessment of evidence, and the proper visualization of data.

## Alternative Models and Prior Distributions: A Sensitivity Analysis

In our modeling efforts we had to make several choices, and it is true that alternative models and alternative prior specifications could be proposed. For instance, one could implement the hierarchical structure using a beta-binomial, and impose an additive structure on the probit-transformed mean of the group-level beta distribution. One could also use a logit transformation instead of a probit transformation, or assume the group-level structure follows a *t* distribution instead of a Normal distribution.

We found that our results are robust against many such changes, although it is impossible to investigate all of the different possibilities. In general, our modeling choices were made for good reasons – for instance, we used the probit transform to stay in the family of generalized linear models, we used the prior on δ for theoretical reasons (i.e., as a unit information prior), we used the uniform prior on σ because of a recommendation by Gelman (2006).

Nevertheless, it is certainly the case that the prior on effect size δ can have a pronounced effect on the Bayes factor. This is understandable; when the prior on δ is highly peaked around the value δ = 0, the hypotheses *H*_0_ and *H*_1_ are actually highly similar. The more similar the competing hypotheses, the more difficult it is for the data to conclusively support one hypothesis over the other. We illustrate this with a sensitivity analysis where we studied the effect that the prior on δ has on the Bayes factor for the surname cities data set. We considered three different priors for effect size δ. As before, we used the unit information prior, *p*(δ) ∼ *N*(0,1). We also considered the “knowledge-based prior,” *p*(δ) ∼ *N*(0, 0.303), a prior proposed by Bem et al. (2011) for the effect of extra-sensory perception; therefore, this prior is a plausible lower bound for the effect sizes expected under the NLE hypothesis. Finally, we considered an in-between prior, namely *p*(δ) ∼ *N*(0, 0.6). We also considered three different priors for the group-level SDs: *p*(σ) ∼ *U*(0, 10), *p*(σ) ∼ *U*(0, 5), and *p*(σ) ∼ *U*(0, 2). We calculated the Bayes factor for all 3 × 3 combinations of priors for δ and σ, both for the unrestricted test of *H*_0_ :δ = 0 against *H*_1_ : δ ≠ 0 and the order-restricted test of *H*_0_ against *H*_2_ : δ > 0. Table 3 shows the results.

**Table 3:** **Results of a sensitivity analysis for the surname cities data set**.

	Prior on effect size δ					
	*N*(0, 0.303)		*N*(0, 0.6)		*N*(0, 1)	
Prior on σ	*BF*_10_	*BF*_20_	*BF*_10_	*BF*_20_	*BF*_10_	*BF*_20_
Uniform (0, 2)	6.51	12.97	25.39	50.73	33.62	67.19
Uniform (0, 5)	6.81	13.57	24.36	48.68	33.08	76.12
Uniform (0, 10)	6.70	13.35	23.91	47.77	37.23	74.42

*The prior on effect size changes the similarity between the null hypothesis and the alternative hypotheses, and therefore the prior affects the diagnostic value of the data (i.e., the Bayes factor). The order-restricted test is based on the renormalization method*.

As is evident from the table, the prior on σ does not exert much of an influence on the Bayes factor. Also, because most of the posterior mass is consistent with the order-restriction, the Bayes factors for the order-restricted tests are about twice as strong in favor of the alternative hypothesis as the Bayes factors for the unrestricted tests. Finally, it is also evident from Table 3 that the prior on δ does have an influence on the Bayes factor: when *p*(δ) ∼ *N*(0, 0.303), the alternative hypothesis is relatively similar to the null hypothesis and this reduces the diagnostic value of the data. Nevertheless, the data support *H*_1_ over *H*_0_ across a range of specifications for δ.

## General Discussion

In this article we have outlined a Bayesian hierarchical test for the analysis of associations between people’s names and their behavior (i.e., the city they live in, the professions they choose, the partners they pursue, the companies they work for, or the products that they buy). The test is easily implemented in WinBUGS (see Appendix) and it allows for coherent inference both on the level of the individual units and on the level of the group.

Our hierarchical analysis strikes an automatic and rational compromise between two existing traditions of analysis, that of complete pooling – in which all names are treated as identical – and that of complete independence – in which every name is treated uniquely. Violin plots show the posterior distribution of the name-letter effect (NLE) for each individual unit (e.g., Figures 2 and 4) and provide a quick overview of the precision and location of the individual-unit NLE. This individual-unit analysis revealed that for several cities there was a clear indication of a reverse NLE, meaning that people gravitate away from cities that resemble their name. We believe this finding may challenge current theories of implicit egotism (see also Gallucci, 2003).

To quantify the evidence for and against a group-level NLE, we used Bayes factors that pitted *H*_0_ against a possibly order-restricted alternative hypothesis. This group-level assessment showed that the Saint city data set did not support a NLE, but the surname city data set did. What are we to conclude from this?^8^ Do people gravitate toward cities that resemble their surnames, but not toward cities that resemble their first names? We do not believe this is a plausible or parsimonious explanation. An alternative explanation is that, even though the Bayes factors suggest a conflict when the two studies are evaluated in isolation, they are in fact consistent; the Saint city data set shows a positive effect size, and so does the surname city data set. In order to assess whether the two data sets are indeed consistent with each other one could either compare the two studies directly (Gelman and Stern, 2006), or, better still, one could collect many similar data sets and then carry out a meta-analysis. Our Bayesian framework can easily be extended to carry out such a meta-analysis; for example, each study *j* could be characterized by a group-level NLE μ*_j_*, and these study-specific NLE’s can be assumed to follow from a higher-level Normal distribution.

On a related note, we feel it is important that researchers interested in the NLE investigate not a single data set, but an entire array of data sets. For instance, it is not convincing when a study shows that, say, people whose surname starts with the letter “A” are disproportionally likely to live in Amsterdam – after all, this result may have been obtained by cherry-picking. It would be much more convincing if the same result hold for all letters, and for most major cities (for an illustration of this important point see McCullough and McWilliams, 2010). The results from these different units may then be combined using our hierarchical Bayesian model.

On *a priori* grounds, some researchers may be skeptical about the impact of name letters on major life decisions. Therefore, the evidence presented in this paper may not be enough to overcome a researcher’s strong prior belief that name letters do *not* influence major life decisions (see also McCullough and McWilliams, 2010, 2011; LeBel and Paunonen, 2011; Simonsohn, 2011a,b,c). These strong prior beliefs do not influence our Bayesian hypothesis test (which is based on the Bayes factor), but they can be incorporated in our statistical framework via the prior model odds *p*(*H*_0_)/*p*(*H*_2_). For example, the surname cities data set yielded a Bayes factor of about 75 in favor of *H*_2_. If the prior model odds are strongly biased against *H*_2_ (e.g., 0.99/0.01 = 99), then a researcher’s posterior model odds may still favor the null hypothesis, albeit less strongly than before.

In sum, our analysis provides a useful novel perspective on the analysis of name-behavior associations in large databases. In addition, our analyses seamlessly carry over to data analysis problems of a similar structure. We believe that hierarchical Bayesian models allow for an assessment of the name-letter effect that is more comprehensive and more coherent than the one that is currently standard.

## Conflict of Interest Statement

The authors declare that the research was conducted in the absence of any commercial or financial relationships that could be construed as a potential conflict of interest.

## Footnotes

^1^We are grateful to an anonymous reviewer of an earlier draft for bringing this to our attention so vividly.

^2^Because the female Saint cities contain few data, we restrict our analyses to the male Saint cities. However, when the female Saint cities are included the results are virtually identical, that is, *BF*_01_ ≈ 2.18 and *BF*_02.M1_ ≈ 1.24, *BF*_02.*M*2_ ≈ 1.29; see the later sections on hypothesis testing.

^3^For more information on WinBUGS see http://www.mrc-bsu.cam.ac.uk/bugs/

^4^Chains that have converged look like fat hairy caterpillars that are completely intermixed.

^5^The fact that *H*_1_ does not contain the single point δ = 0 is inconsequential because δ is a continuous parameter – inference is therefore unaffected by including or excluding δ = 0 from *H*_1_

^6^For continuous distributions the definition of probability involves the area under the curve, not the height of the curve at a particular point. Therefore, the density values can exceed 1.

^7^A Bayesian confidence interval is often called “credible interval,” but here we retain the standard terminology.

^8^A reviewer on an earlier draft felt that these conflicting conclusions showed that Bayesian inference has failed. We disagree: the method of Bayesian inference cannot be held accountable for the quality or consistency of the data with which it is confronted.

## Appendix

### WinBUGS code for the saint cities and the surname cities

This appendix provides the model specification code that implements the graphical model shown in Figure 1 in WinBUGS. For our analyses, we called WinBUGS from R using the R2WinBUGS package (Sturtz et al., 2005). R is a free software distributed under the GNU license (R Development Core Team, 2008). Some calculations are done in R before calling WinBUGS:

1. Transform the baseline proportion *b*_*i*_ (proportion of people with a particular name deceased in the U.S.) to the probit scale.
  β_*i*_ = Φ^−1^(*b*_*i*_), in R: beta = qnorm(b)
2. Calculate the number *N*_*i*_ of people with a particular name deceased in the respective city from “proportion in city” and “city population” *TN*_*i*_ and round to integers.
  *N**_i_* = proportion in city_i_ × *TN**_i_*, in R: N = round((N.prop * TN), digits = 0)
3. Introduce a variable *M* that indicates the number of independent replications (the part enclosed by the rounded rectangle in Figure 1). For the Saint cities data set, there are 27 cities, thus *M *= 27. For the surname cities data set, there are 30 cities, thus *M *= 30.
  In R: M = length(beta) sets *M* equal to the number of elements in the vector “beta”.

In the WinBUGS code below, the twiddle symbol “∼”means “is distributed as” and the hash sign “#” is used for comments. Note that in WinBUGS, a Normal distribution is specified in terms of mean and precision (i.e., the inverse of the variance).

model
{
 for(i in 1:M)
 {
 # number of people with particular name in
 # name-resembling city is binomially distributed:
 N[i] ˜ dbin(theta[i], TN[i])
 # probit transformation of rate parameter:
 theta[i] <− phi(gamma[i])
 # probitized rate parameter = probitized baseline + NLE:
 gamma[i] <− beta[i] + alpha[i]
 # NLEs for individual cities drawn from a
 # group-level Gaussian distribution:
 alpha[i] ˜ dnorm(mu, lambda)
 }
 # GROUP-LEVEL PARAMETERS:
 # mean mu is defined in terms of effect size delta and
 # standard deviation sigma
 mu <− delta * sigma
 # precision lambda is the inverse of the variance:
 lambda <− pow(sigma, −2)
 # uniform prior from 0 to 10 on sigma:
 sigma ˜ dunif(0,10)
 # standard Normal prior on effect size delta:
 delta ˜ dnorm(0,1)
}

For the analysis of both the Saint city data set and the surname city data set, the above WinBUGS code was used to generate five MCMC chains, each comprised of 50,000 iterations; after discarding the first 20,000 iterations from each chain as burn-in and confirming convergence by visual inspection and the $\hat{\text{R}}$ statistic (Gelman and Rubin, 1992), we collapsed the samples across the five chains so that our inference was based on a total of 150,000 samples from the joint posterior.
